# Revision of Carpal Tunnel Surgery

**DOI:** 10.3390/jcm11051386

**Published:** 2022-03-03

**Authors:** Stahs Pripotnev, Susan E. Mackinnon

**Affiliations:** 1 Plastic and Reconstructive Surgery, Roth|McFarlane Hand and Upper Limb Centre, Western University, London, ON N6A 4V2, Canada; stahspripotnev@gmail.com; 2 Plastic and Reconstructive Surgery, Washington University School of Medicine, Barnes Jewish Hospital, St. Louis, MO 63110, USA

**Keywords:** carpal tunnel syndrome, neuropathy, revision carpal tunnel release, neurolysis

## Abstract

Carpal tunnel release is one of the most commonly performed upper extremity procedures. The majority of patients experience significant improvement or resolution of their symptoms. However, a small but important subset of patients will experience the failure of their initial surgery. These patients can be grouped into persistent, recurrent, and new symptom categories. The approach to these patients starts with a thorough clinical examination and is supplemented with electrodiagnostic studies. The step-wise surgical management of revision carpal tunnel surgery consists of the proximal exploration of the median nerve, Guyon’s release with neurolysis, the rerelease of the transverse retinaculum, evaluation of the nerve injury, treatment of secondary sites of compression, and potential ancillary procedures. The approach and management of failed carpal tunnel release are reviewed in this article.

## 1. Introduction

Carpal tunnel syndrome (CTS) is the most common peripheral nerve compression syndrome with prevalence rates varying between 1–5% of the general population resulting in approximately 600,000 carpal tunnel releases per year in the United States [1,2,3,4,5,6,7]. Open carpal tunnel release (CTR) remains the gold standard procedure of choice but alternative techniques including limited incisions and endoscopic release have also been described [8,9,10,11]. A recent paper by Westenberg et al. (2020) identified a revision rate of 1.5% in a large group of 7464 patients that underwent carpal tunnel release from 2002–2015. They found that male sex, rheumatoid arthritis, smoking, endoscopic release, and simultaneous bilateral releases to be risk factors for increased risk of revision [12]. This review will describe the etiology of failed CTR, the approach to these patients, and the reconstructive options available.

## 2. Etiology of Failed Carpal Tunnel Release

Although CTR is associated with significant improvement of symptoms and satisfaction in the majority of patients, a small but important subset will experience ongoing or recurring symptoms. We group these patients into three primary categories—persistent, recurrent, and new symptoms [13].

### 2.1. Persistent Symptoms

#### 2.1.1. Incomplete Release

**Figure 1:**
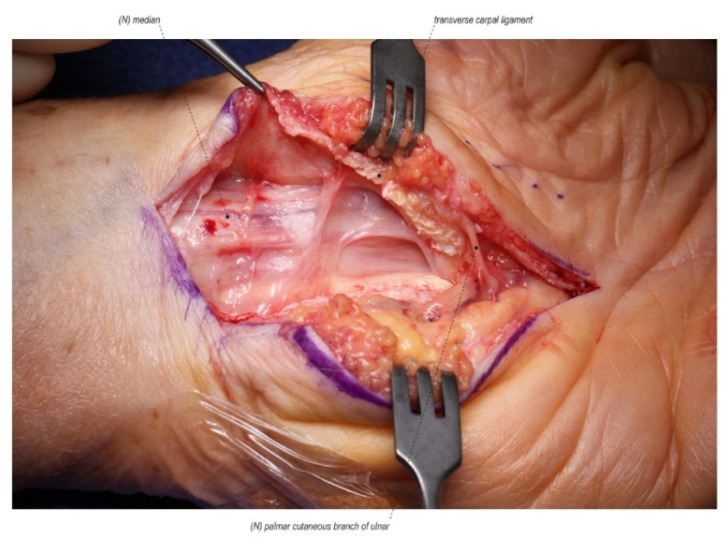
A scarred median nerve adherent to the undersurface of the radial leaf of the transverse carpal ligament in a case of a revision open carpal tunnel release. Proximal nerve bruising and swelling confirms ongoing compression despite the previous release. (Left hand, proximal to the left).

The most common reason for persistent symptoms after CTR is the incomplete release of the transverse carpal ligament (Figure 1). This incomplete release can occur distally at the palmar arch or proximally at the volar forearm antebrachial fascia which can be especially thickened in patients who develop carpal tunnel syndrome as a result of trauma. The incomplete release is likely the result of inadequate exposure and visualization [12,13,14,15,16].

#### 2.1.2. Secondary Sites of Compression

**Figure 2:**
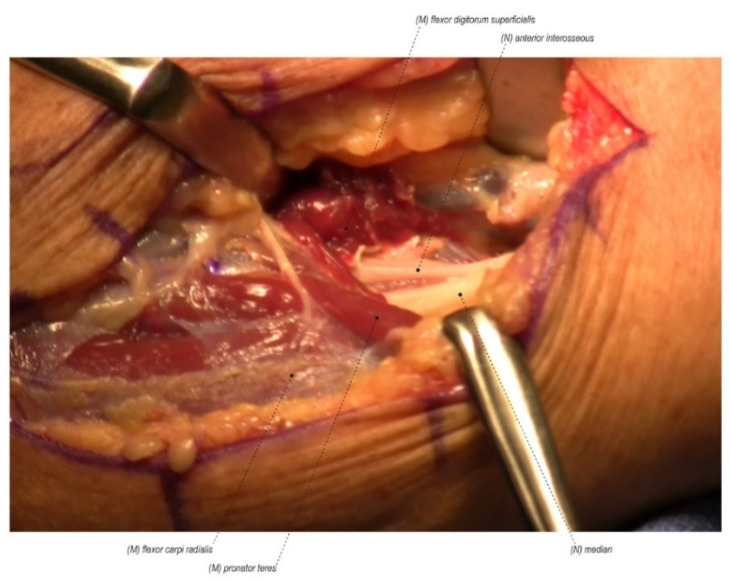
Pronator syndrome consists of median nerve compression more proximally in the forearm at the ligament of Struthers, the bicipital aponeurosis (Lacertus fibrosis), the flexor digitorum superficialis arch, or between the two heads of the pronator teres. (Right forearm, proximal to the right).

Another reason for persistent symptoms after CTR is failure to identify a secondary site of compression in addition to the primary compression at the carpal tunnel. Although the carpal tunnel is the most common site of compression for the median nerve, it can also be compressed more proximally in the forearm at the ligament of Struthers, the bicipital aponeurosis (Lacertus fibrosis), the flexor digitorum superficialis arch, or between the two heads of the pronator teres. This compression entity has been termed pronator syndrome. We describe it more inclusively as median nerve compression in the forearm (MNCF) (Figure 2) [17,18,19,20,21,22,23]. CTS is also well known to be associated with radiculopathy in the cervical spine due to a double crush phenomenon [24,25,26,27]. Failure to recognize a secondary site of compression can lead to incomplete resolution of symptoms.

#### 2.1.3. Wrong Diagnosis

Lastly, persistent symptoms after CTR can be due to an initially wrong diagnosis. In the same manner, as a secondary site of compression, the alternative site of compression can also be the primary source of the symptoms with no contribution from the carpal tunnel. Alternatively, similar symptoms can occur in median nerve tumors and in a variety of other unrelated upper extremity pathologies [16,28,29]. A thorough and proper history and physical exam are critical to the accurate diagnosis of CTS [30,31].

### 2.2. Recurrent Symptoms

#### 2.2.1. Scar Formation

The most common cause of recurrent symptoms returning after a period of symptom relief is due to scar formation in the carpal tunnel post-operatively. Typically, the scar is between the median nerve and an incision placed directly above the nerve leading to tethering of the nerve and recurrent compression, and in some cases reformation of a pseudo transverse carpal ligament (Figure 1) [32,33,34,35]. Increased scar formation can be attributed to prolonged immobilization, poor hemostasis, hematoma formation, or inappropriate hand therapy [13]. Tenosynovitis associated with rheumatoid arthritis or certain conditions such as amyloidosis can also predispose patients to recurrent symptoms [36,37,38].

#### 2.2.2. Secondary Sites of Compression

Similar to how a secondary site of compression can lead to persistent symptoms after CTR, these additional sites of compression can also manifest later as recurrent symptoms.

### 2.3. New Symptoms

#### 2.3.1. Iatrogenic Injury

A patient that complains of new painful symptoms and neurological loss immediately or shortly after carpal tunnel release may have symptoms related to acute ischemic injury from an incomplete release. By contrast, especially with severe pain, they may have an iatrogenic neurotmetic injury. Injury can occur to the palmar cutaneous nerve, palmar cutaneous branches of the ulnar nerve, digital nerves, or even the median and ulnar nerves themselves [39,40,41,42,43]. Injury to the palmar cutaneous can be avoided with a more ulnarly-positioned incision which can be placed in the watershed zone between the median and ulnar-based palmar cutaneous nerves [44]. The third webspace branch of the median nerve is especially vulnerable to nerve injury at the distal aspect of the release.

#### 2.3.2. Secondary Pathology

Another final group of patients will have new symptoms related to entirely new pathology which can be related or unrelated to the carpal tunnel release. Primary compression of the palmar cutaneous branch has been reported and post-operative swelling after a CTR can precipitate this alternative compression neuropathy since the palmar cutaneous branch passes through its own tunnel [45]. Carpal tunnel release has also been associated with subsequent carpal arch alterations, Pisotriquetral Syndrome, and flexor tendon bowstringing [46,47,48,49].

## 3. Approach and Assessment

The approach to patients with failed carpal tunnel release starts with a thorough history and physical examination [30,31]. Simply inquiring about the timing surrounding the presenting complaint can help categorize patients into one of the aforementioned groups. If a patient reports that the symptoms never resolved and possibly even worsened after the release then an incomplete release, secondary compression, or alternate diagnosis should be considered. If a patient reports that their symptoms resolved but returned at a later date then scar formation or a secondary pathology is investigated. Lastly, if a patient reports a new pain and/or motor weakness after the procedure then an iatrogenic injury is suspected. If a pain diagram was collected preoperatively then it can be used to compare the current severity and distribution of symptoms.

In addition to standard upper extremity musculoskeletal and neurologic examination, specific maneuvers and provocative tests can help further delineate symptom etiology. Patients with persistent or recurrent symptoms should be examined for ongoing compression at the carpal tunnel with Durkan’s, Phalen’s, or other similar provocative tests [50,51]. Pronator syndrome can be assessed as a secondary site of compression with techniques to provoke symptoms such as maximal supination and simultaneous pressure at the proximal volar forearm [17,18,19,20]. The sensory collapse test can also be used to parse out the hierarchy of compression using ethyl chloride spray and can even determine if the carpal tunnel release is incomplete proximally at the antebrachial fascia or distally in the palm [52,53]. Patients with new pain should be assessed for iatrogenic injury with the Tinel sign just proximal to the suspected injured nerve [51,54,55]. Visually, the location and length of the scar can also provide clues as to which nerve may be injured. Each of the eight grouped fascicular components of the median nerve is tested using the ten test, two-point discrimination, Semmes Weinstein monofilament, and motor examination [56]. This is key with iatrogenic injuries.

A preoperative electrodiagnostic study (EDX) is very useful to compare with repeat EDX especially if there is worsening. A sudden significant loss of sensory nerve action potentials (SNAP), compound muscle action potentials (CMAP), or motor unit action potentials (MUAP) suggests an acute nerve injury [27,57,58,59]. New electrodiagnostic findings in the ulnar innervated territory can indicate an inadvertent ulnar nerve injury. Electrodiagnostics can also be used to assess for cervical radiculopathy as an alternate or secondary site of compression contributing to the symptoms. It is important to note that in patients with moderate-severe CTS, EDX findings will not return to normal even with complete relief of symptoms. High-resolution ultrasound can be used to identify potential structural causes of recurrence [60,61,62]. Carita et al. imaged 35 median nerves with recurrent symptoms and found persistent compression in 30 out of the 35 with incomplete release in 20, perineural fibrosis in four, both of these findings in five, and tenosynovitis in one patient. In the remaining five patients, four had evidence of nerve injury and one had no abnormalities [60]. As MRI technology improves, there is growing evidence that it can also be used to confirm the presence of incomplete release or recurrence due to scar formation [63,64,65]. Campagna et al. found a significant difference in fibrosis, median nerve enhancement, and nerve width when two blinded observers reviewed the 1.5 T MRIs of 35 recurrent cases compared to 12 controls [63].

## 4. Management

As with any revision operation, there are increased risks and complexities due to scarring and an element of uncertainty as to what will be found upon re-exploration of the surgical field. Surgeons undertaking revision carpal tunnel release should be prepared for a variety of findings with multiple techniques at their disposal to achieve a successful outcome. Although primary carpal tunnel release can be safely performed under local anesthesia, due to the increased complexity of revision cases, we perform all revision cases under general anesthesia in the main operating room for better lighting, equipment, and prolonged tourniquet time.

### 4.1. Repeat Carpal Tunnel Release

The first step to any revision carpal tunnel release is to identify the normal median nerve in the forearm well proximal to the carpal tunnel. It is our preference to place the revision incision ulnar to the previous incision and cross the wrist into the forearm in a Bruner fashion in order to dissect through unscarred tissue as much as possible. Therefore, in addition to the ulnar translation, the revision incision is much longer than the original and should cross the wrist crease in order to identify the median nerve proximally prior to tracing it through the previous surgical site. The second step is to open Guyon’s canal to expose and release the flexor retinaculum on the ulnar side. Traction on the retinaculum will typically show that the median nerve is adherent to the previous incision. With this combination of a proximal and ulnar approach, the median nerve can then typically be found adherent to the undersurface of the radial leaf of the transverse carpal ligament (Figure 1) [13].

There is some literature on endoscopic and ultrasound-guided approaches to revision cases. Luria et al. described improvement in 37 out of 41 revision endoscopic carpal tunnel releases [66]. Fried and Nazarian described case reports of ultrasound-guided hydroneurolysis of the median nerve in recurrent carpal tunnel syndrome with good success [67]. It is our opinion that although these articles report good results, there are significant limitations to these techniques such as the inability to manage previous nerve injury or perform neurolysis, and the potential risk of new injury to the median nerve due to tethering secondary to scar tissue. Our preferred approach is an extended open release.

### 4.2. External and Internal Neurolysis

**Figure 3:**
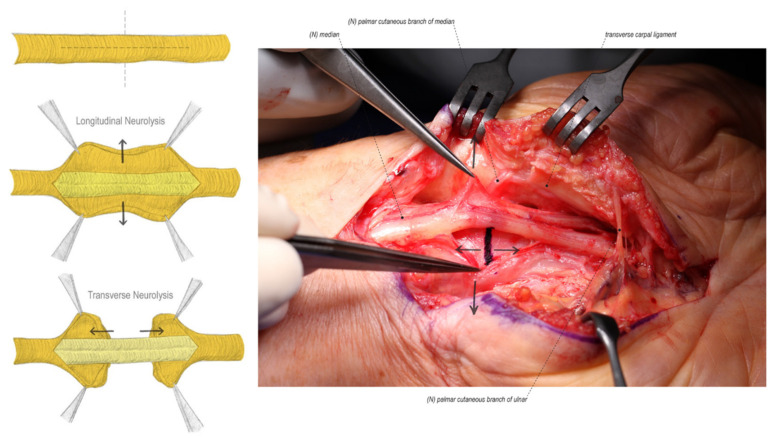
External neurolysis consists of both longitudinal and transverse epineurium release until bands of Fontana can be visualized on the nerve fascicles. (Left hand, proximal to the left).

Once the median nerve is safely identified, a neurolysis is performed until healthy fascicular structure and bands of Fontana can be visualized (Figure 3) [13]. External neurolysis has been well proven to be beneficial in recurrent symptom resolution by separating the nerve from the surrounding scar [34,68,69]. Duclos et al. published a series of 13 patients and found complete resolution of symptoms in 75% of patients after repeat exploration and external neurolysis [34]. Described by Curtis and Eversmann, and subsequently supported by Rhoades et al. and Wagstroem and Nigst, internal neurolysis has been shown to be safe [70,71,72]. While further research by Gelberman et al. and Mackinnon et al. showed that neurolysis is not beneficial in primary CTS [73,74]. Neurolysis is a graded procedure ranging from external epineurotomy to internal neurolysis continuing in a progressive fashion based on the degree of fibrosis present. We perform a longitudinal and transverse external epineurotomy and progress until fascicles are identified and bands of Fontana are seen [13].

### 4.3. Neuroma or Nerve Injury Identification and Management

**Figure 4:**
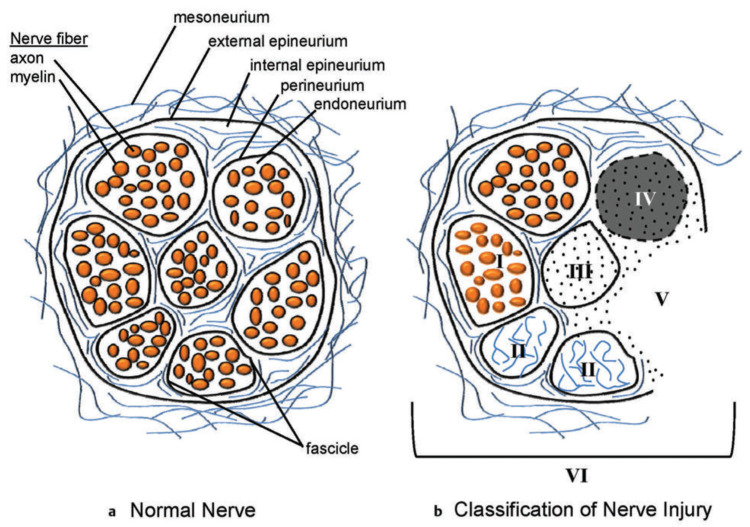
Peripheral nerve organization and injury classification. (**a**) Schematic representation of the cross-section of a normal peripheral nerve showing the connective tissue and nerve tissue components. (**b**) The cross-section of the peripheral nerve demonstrates a mixed, or sixth-degree, injury pattern. The fascicle at the top left is normal. Moving in the counterclockwise direction, fascicle I is a first-degree injury (neurapraxia) with segmental demyelination. Fascicle II is a second-degree injury (axonotemesis). The second degree involves both the axon and the myelin. The endoneurial tissue is not damaged. Fascicle III demonstrates a third-degree injury, with injury to the axon, myelin, and endoneurium. The perineurium is intact and normal. Fascicle IV demonstrates a fourth-degree injury, with injury to the axon, myelin, endoneurium, and perineurium. The fascicle is marked by scarring across the nerve, with only the epineurium being intact. Fascicle V is a fifth-degree injury in which the nerve is not in continuity and is transected. The surgeon will separate the fourth- and fifth-degree injury patterns, which will require reconstruction from the normal fascicles and the fascicles demonstrating first-, second-, and third-degree injury patterns (such latter patterns of injury require, at most, neurolysis). (Reprinted with permission from Mackinnon SE. Nerve Surgery. New York, NY, USA: Thieme; 2015). Note: A Sunderland zero injury would be illustrated as a normal fascicle with no blood vessels.

**Figure 5:**
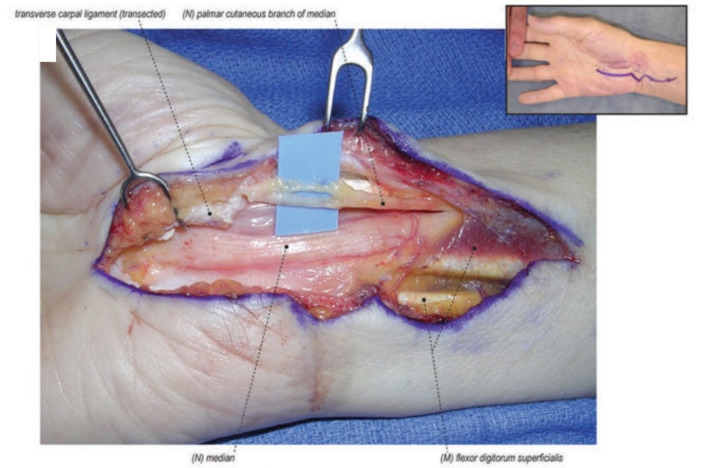
A palmar cutaneous nerve neuroma in a revision carpal tunnel release. (Right hand, proximal to the right).

The next step in a revision carpal tunnel release is to assess for evidence of nerve injury. This is particularly important in a patient who had new pain after the primary release suggesting the possibility of an iatrogenic injury. The main median nerve (and ulnar nerve if an injury was suspected clinically) is examined during the neurolysis. The nerve injury is treated as a Sunderland 6th degree injury with individual fascicles managed separately (Figure 4) [75,76,77,78]. Preoperative evaluation coupled with EDX is helpful in predicting intraoperative findings. Under loupe microsurgical magnification, the neurotmetic injured fascicles are trimmed back to healthy nerve and reconstructed with interposition nerve autograft. For short distances, the lateral antebrachial cutaneous nerve (LABC) can be harvested in the proximal forearm where it lies adjacent to the cephalic vein just medial to the brachioradialis muscle. For longer nerve graft segments, we prefer to use the medial antebrachial cutaneous nerve (MABC) which can be found traveling with the basilic vein medial to the biceps in the upper arm. Advantages of these alternative donors over the commonly selected sural nerve include confining donor morbidity to the affected limb, limiting additional incisions, speed and simplicity of harvest, and the ability to repair the distal end of the injured nerve to the side of a nearby intact nerve as a nerve transfer to recover sensation into the donor territory (Figure 5) [79,80,81,82,83].

**Figure 6:**
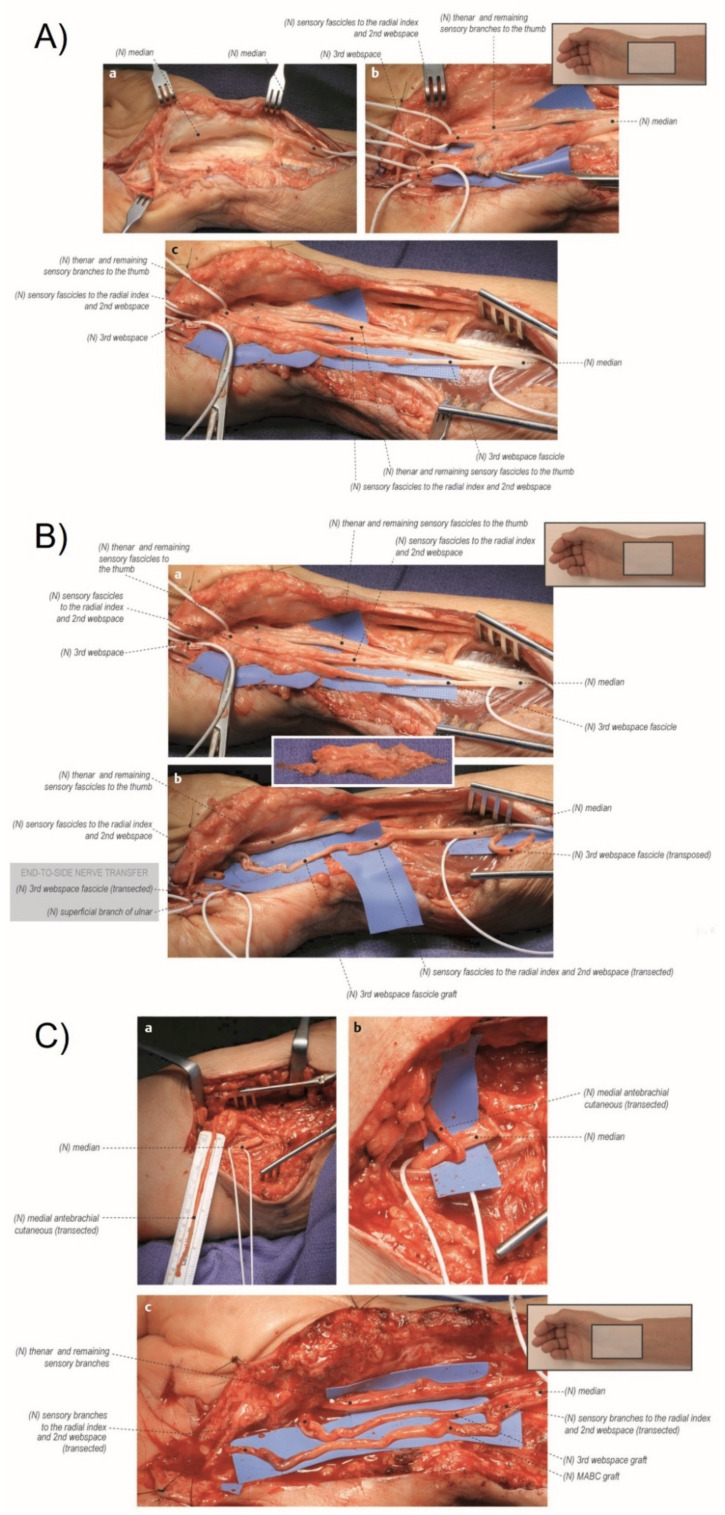
(**A**) Exposure of the right median nerve and internal neurolysis. (a) The median nerve was identified proximal and distal to the zone of injury. It was found to have a course within dense scar tissue. (b) The median nerve was isolated from the scar tissue, and distal neurolysis revealed the sensory branches of the median nerve. The intact thenar motor branch and sensory fascicles to the thumb were protected. Suture material was found within the remainder of the injured median nerve. (c) Proximal neurolysis revealed the fascicular anatomy of the median nerve. The third web space is neurolyzed proximally so that it can be used as a graft material. (**B**) Neuroma resection and first web space grafted with a third web space graft. (a) The zone of injury was identified, and the neuroma was resected with proximal and distal median nerve components identified. The third web space was further neurolyzed proximally to mobilize graft material. (b) The proximal end of the third web space fascicle was transected and used as a nerve graft to repair a portion of the median nerve. The proximal remainder of the third web space was transposed proximally to prevent a painful neuroma. The distal third web space was end-to-side transferred to the sensory component of the ulnar nerve to provide a rudimentary sensation for donor deficit. (**C**) Second web space grafted with a medial antebrachial cutaneous nerve (MABC) graft. (a) The MABC was isolated within the arm for donor material. (b) The MABC was then transected with the distal end transferred to the sensory component of the median nerve through an end-to-side epineural window fashion. The sensory component of the median nerve is located on the superior aspect of the median nerve. Note that, in this image, the median nerve has been rotated so that it appears to be on the inferior portion. (c) The MABC graft was used to repair the remaining portion of the median nerve. The thenar branch and remaining sensory branches to the thumb were protected and were found to be not injured. (Reprinted with permission from Mackinnon SE. Nerve Surgery. New York, NY, USA: Thieme; 2015).

The palmar cutaneous branch is also identified and examined for signs of injury. If a neuroma is identified or there is high enough suspicion from the preoperative exam for a nerve injury then the palmar cutaneous nerve is neurolyzed proximally from the median nerve until there is an adequate length for proximal transposition and burial between the superficial and deep flexors of the forearm. The neuroma is excised, the end of the nerve is capped with electrocautery and a proximal crush is applied to the nerve with a hemostat to reset the site of the nerve injury more proximally (Figure 6). This combination of techniques minimizes the chances of painful neuroma recurrence [84,85,86].

### 4.4. Nerve Coverage

The final step in a revision carpal tunnel release is considering the need for median nerve coverage with vascularized tissue or commercial nerve barriers. In patients with persistent symptoms due to incomplete release, a revision with external neurolysis alone may be sufficient. However, in recurrent cases, the rationale behind soft tissue coverage is that unless the surrounding tissue environment is changed, then the pathologic process that led to recurrence will once again repeat itself. The potential advantages of vascularized tissue coverage include increased blood flow to the median nerve which may promote axonal regrowth, reducing the possibility of overlying dysesthetic skin reinnervation, creating a favorable gliding plane, and mechanical interposition between the edges of the transverse carpal ligament to prevent reformation [35,86,87,88].

### 4.5. Author’s Preference

**Figure 7:**
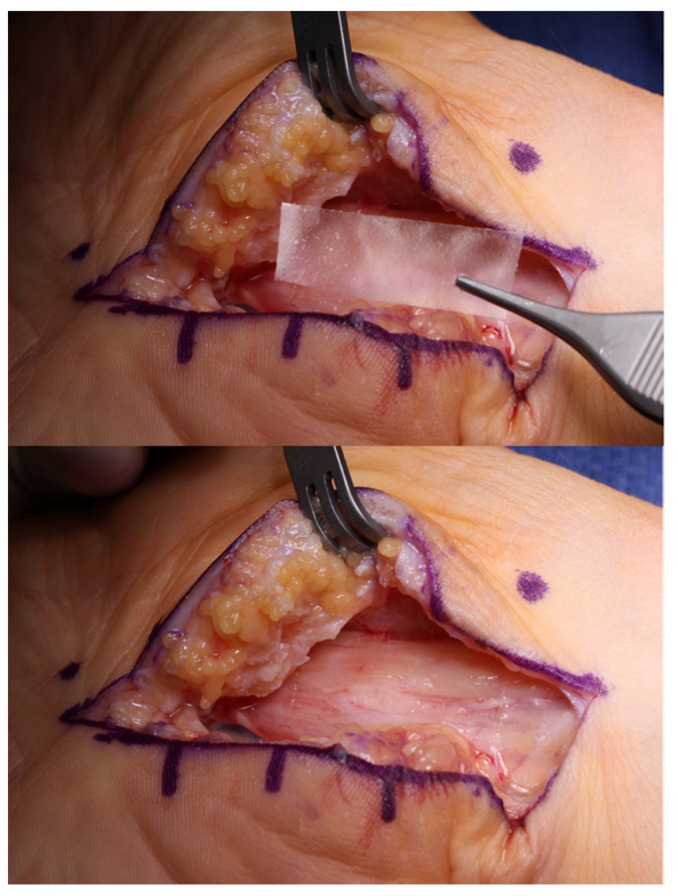
Hyaluronic acid-carboxycellulose membrane (Seprafilm) can be placed over the median nerve after a revision open carpal tunnel release to reduce post-operative scarring and recurrent adhesion formation. (Right hand, proximal to the right).

Our preferred choice of nerve coverage has evolved over the years from using the pronator flap to the hypothenar fat pad flap, and now with the use of hyaluronic acid-carboxycellulose membrane (Seprafilm) placed superficially on the nerve without circumferential wrapping (Figure 7). Although some authors have found success with the circumferential wrapping of nerves, based on our experience with removing previously wrapped products, there can be significant local ischemia on the nerve due to the wrap [89]. We observe that the wrapped product succeeds in reducing external scarring at its external surface, but the wrap itself creates a dense constrictive scar around the nerve when placed circumferentially [90,91].

### 4.6. Comprehensive Review of Nerve Coverage Options

Wulle described the synovial flap which utilizes the flexor tendon synovial tissue for median nerve coverage. He showed six excellent, 16 good, three satisfactory, and two bad results in a series of 27 patients [92]. There are several local muscle flaps described for median nerve coverage including the lumbricals as described by Koncilia et al. [93], the abductor digiti minimi described by Milward et al. [94], the pronator quadratus described by Mackinnon and Dellon [95], and the palmaris brevis described by Rose et al. [88]. A final common local flap of choice is the hypothenar fat pad flap which was first described for revision carpal tunnel surgery by Strickland et al. [96] It can be harvested within the same carpal tunnel release exposure by elevating the ulnar sided skin off the hypothenar fat and mobilizing the fat pad radially based on the small ulnar artery perforators that supply it. It is then inset to the radial leaf of the transverse carpal ligament [97]. Strickland et al. showed resolution of symptoms in 89% of patients and Mathoulin et al. showed resolution in 94% of patients [96,97]. All of these flaps provide a small vascularized segment of tissue with minimal donor morbidity. Although there are series describing good outcomes for all of them, there are no high-level comparative or randomized studies to support the use of one over another. Additionally, given the heterogeneous nature of revision carpal tunnel patients, such a study would not be feasible. Therefore, the choice of local flap coverage is up to the surgeon based on their training and preference. Lastly, larger regional flaps such as the reverse radial forearm or even free flaps have been described but these should be reserved for severe recalcitrant cases [15,98].

There are also several commercial products designed to provide a barrier and reduce perineural scarring. This includes products composed of bovine collagen (NeuraWrap), porcine small intestine submucosa (AxoGuard), and hyaluronic acid-carboxycellulose membrane (Seprafilm) [99]. In a rat sciatic nerve model, Lee et al. found a significant reduction in perineural scarring in the group that was treated with suture repair and collagen conduit compared to just suture repair alone [100]. Kokkalis et al. published a two-patient case series where both patients had resolution of their symptoms after revision carpal tunnel release and treatment with a collagen wrap [101]. Similarly, Soltani et al. found an 89% subjective positive response in nine revision carpal tunnel patients and an 83% positive response in six revision cubital patients treated with a collagen nerve wrap [102]. Additionally, in a rat sciatic model, Magill et al. showed qualitatively less perineural scar formation with no deleterious effects when sciatic nerve repairs were wrapped with Seprafilm [103]. Despite these positive results, the use of nerve wraps remains controversial due to the lack of strong comparative studies proving their efficacy. It is our preference to avoid all circumferential nerve barriers due to the potential for nerve constriction as seen based on clinical experience [90,91].

### 4.7. Secondary Sites of Compression

Based on the preoperative clinical exam, decompression of secondary sites of compression may be required. Median nerve decompression in the forearm is accessed through a volar forearm incision. The Lacertus fibrosis is released and the pronator teres tendon is step lengthened and then the median nerve is decompressed by fully releasing the deep head of the pronator teres and the leading edge of the flexor digitorum superficialis arch. Rarely, the palmar cutaneous nerve can be compressed in its own tunnel and only requires a decompression [45].

### 4.8. Ancillary Procedures for Chronic Disease

Patients who suffer prolonged chronic and recurrent carpal tunnel syndrome, or those who suffer a median nerve iatrogenic injury, will potentially have a poor predicted outcome with regards to recovery of sensation and motor function. Thus, secondary procedures to help restore that function should also be considered.

Sensory recovery can be supplemented with side-to-side ulnar to median nerve allografts. Felder et al. showed that out of 24 patients that underwent median to ulnar side-to-side sensory grafting for severe ulnar neuropathy, 21 (87%) had the return of protective sensation, 16 (66.7%) had the return of diminished light touch sensation, and six (25%) had the return to normal range sensation within 1 year as assessed by SWMT and/or 2-point discrimination [104]. The same principle can be applied to severe median neuropathy as well. The grafts are placed distal to the site of maximal compression and if allografts are used, the length is kept under 2.5 cm and a width of 2–3 mm is used.

**Figure 8:**
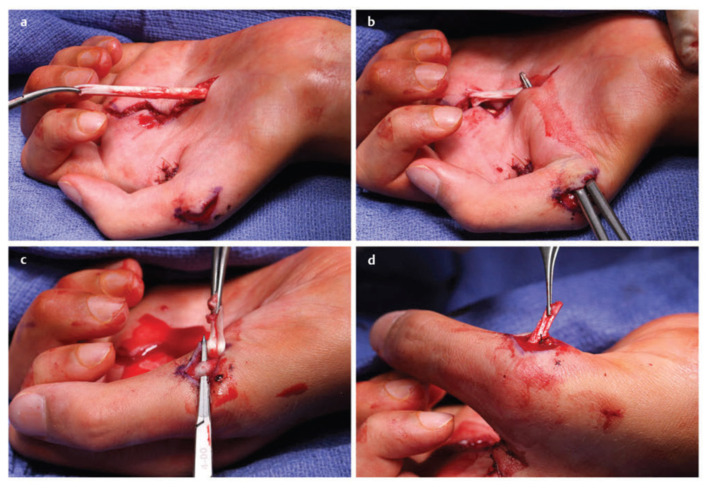
Left flexor digitorum superficialis (FDS) (ring)-to-abductor pollicis brevis (APB) tendon transfer. (**a**) The FDS tendon of the ring finger is harvested distally. (**b**) The FDS ring tendon is tunneled through the subcutaneous fascia toward the APB. (**c**) The tendon of the APB is identified. (**d**) The FDS ring tendon is sutured into the tendon of the APB. Similarly, the flexor carpi ulnaristendon can be used as a pulley. (Reprinted with permission from Mackinnon SE. Nerve Surgery. New York, NY, USA: Thieme; 2015).

**Figure 9:**
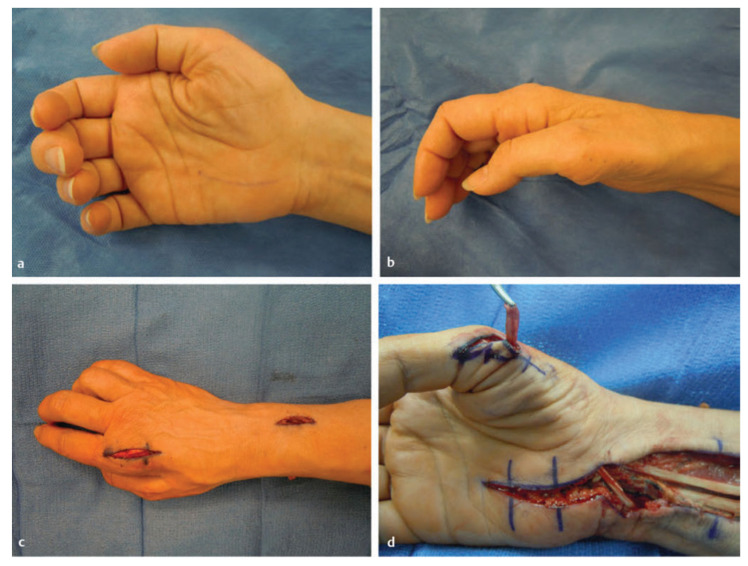
Atrophy of thenar muscles. (**a**,**b**) This patient had a right median nerve injury that is evident with the atrophy of the thenar muscles. Extensor indicis proprius (EIP) opponensplasty: (**c**) Incision for the harvest of the distal EIP tendon. (**d**) The tendon is routed in the posterior/ulnar direction and tunneled through the subcutaneous fascia to the abductor pollicis brevis. (Reprinted with permission from Mackinnon SE. Nerve Surgery. New York, NY, USA: Thieme; 2015).

The motor deficit of chronic carpal tunnel syndrome primarily consists of thenar wasting and weakness with thumb opposition. There are several tendon transfers described for reconstructing thumb opposition including the abductor digiti minimi (Huber) [105], extensor indicis proprius (Burkhalter) (Figure 8) [106,107], flexor digitorum superficialis (Figure 9) [108,109], and the palmaris longus (Camitz) transfer [110,111,112]. Although there is no literature supporting the superiority of any of these transfers compared to the others, the Camitz palmaris longus transfer is most commonly selected (out choice as well) for severe carpal tunnel syndrome because of its simplicity, minimal donor morbidity, and access through the same revision carpal tunnel exposure. A systematic review by Rymer and Thomas showed improvement in hand function in 86–100% of patients that underwent a Camitz transfer [110]. One of the criticisms of the original Camitz transfer is that it does not make use of a pulley and thus only provides thumb abduction due to its direct line of pull [112]. In order to improve on this limitation, several modifications have been described incorporating a pulley [110,113].

## 5. Conclusions

Carpal tunnel release is one of the most commonly performed upper extremity procedures. The majority of patients experience significant improvement or resolution of their symptoms. However, a small but important subset of patients will experience the failure of their initial surgery. These patients can be grouped into persistent, recurrent, and new symptom categories. The approach to these patients starts with a thorough clinical examination and can be supplemented with electrodiagnostic studies and MRI. The step-wise surgical management of revision carpal tunnel surgery consists of a careful re-exploration of the median nerve, neurolysis (external and internal as needed), evaluation of the nerve injury, possible soft tissue coverage, treatment of secondary sites of compression, and potential ancillary procedures.
